# Local Adaptation of Sun-Exposure-Dependent Gene Expression Regulation in Human Skin

**DOI:** 10.1371/journal.pgen.1006382

**Published:** 2016-10-19

**Authors:** Ryosuke Kita, Hunter B. Fraser

**Affiliations:** Department of Biology, Stanford University, Stanford California; University of Pennsylvania, UNITED STATES

## Abstract

Sun-exposure is a key environmental variable in the study of human evolution. Several skin-pigmentation genes serve as classical examples of positive selection, suggesting that sun-exposure has significantly shaped worldwide genomic variation. Here we investigate the interaction between genetic variation and sun-exposure, and how this impacts gene expression regulation. Using RNA-Seq data from 607 human skin samples, we identified thousands of transcripts that are differentially expressed between sun-exposed skin and non-sun-exposed skin. We then tested whether genetic variants may influence each individual’s gene expression response to sun-exposure. Our analysis revealed 10 sun-exposure-dependent gene expression quantitative trait loci (se-eQTLs), including genes involved in skin pigmentation (*SLC45A2*) and epidermal differentiation (*RASSF9*). The allele frequencies of the *RASSF9* se-eQTL across diverse populations correlate with the magnitude of solar radiation experienced by these populations, suggesting local adaptation to varying levels of sunlight. These results provide the first examples of sun-exposure-dependent regulatory variation and suggest that this variation has contributed to recent human adaptation.

## Author Summary

Varying levels of sun-exposure across the world have significantly shaped human evolution. Previous analyses have found several skin pigmentation genes with evidence of strong evolutionary pressures throughout human history, manifesting as large differences in the frequency of genomic variants across populations. But even within populations, individuals respond differently to sun-exposure, suggesting variation in addition to the major differences in skin pigmentation across populations. Here we investigated whether genetic variants associate with response to sun-exposure within Europeans. To measure the response we analyzed gene expression in sun-exposed and non-sun-exposed skin, and identified ten genetic variants that associated with the sun-exposure response of nearby genes. One of these genetic variants, which associated with the sun-exposure response of the gene *RASSF9*, showed evidence of adaptation in humans in response to solar radiation. Together this evidence suggests that the regulation of gene expression is influenced by sun-exposure and that the sun-exposure dependent effect on *RASSF9* expression may have had an effect on human fitness. To our knowledge, this is the first example of an environment-dependent regulatory variant with evidence of adaptation in humans.

## Introduction

Despite the many successes of genome-wide association studies (GWAS), the field of human genetics is still only scraping the surface of questions encountered in day-to-day life. Questions such as why individuals respond differently to sun exposure, diet, or drugs highlight the prominent role of the environment’s varied effects on every individual. When environmental and genetic variation modify one another’s effects on phenotypes, this is known as gene-by-environment, or GxE, interaction. Many classic examples of GxE interactions exist, such as the inherited condition xeroderma pigmentosa resulting in extreme UV sensitivity [1–5]. But because the environment is both unbounded and fluid, the potential number of GxE interactions is infinite, which has hindered progress on basic questions such as the importance of GxE interactions in evolution and disease [6,7]. Indeed, GxE interactions may help explain the “missing” broad-sense heritability that has been the Achilles heel of GWAS [8–10].

Identifying new GxE interactions from genome-wide approaches is challenging due to their typically small effect sizes coupled with a formidable multiple-hypothesis testing burden [11,12]. However these challenges can be overcome by using gene expression as the trait, i.e. studying how the combination of genetic and environmental variation affects gene expression [13]. This is because cis-regulatory variants (also called cis-acting expression quantitative trait loci, or cis-eQTLs) often have large effect sizes, and identification of these cis-eQTLs requires testing only the SNPs nearby a given gene, resulting in a smaller multiple-hypothesis testing burden [14–16]. In addition, the data required to identify GxE interactions affecting gene expression regulation (hereafter referred to as GxE expression variants) are being produced at an ever-accelerating pace, in large part by consortia such as GTEx [17]. Several studies have successfully identified GxE expression variants in model organisms and in humans [18–26]. The human studies [23,24,26–29] have investigated the roles of various external immunological stimuli, statin exposure, and ionizing radiation in GxE interactions. These studies have identified a wealth of GxE expression variants associated with health-related conditions, such as Crohn's disease and drug-induced myotoxicity, suggesting the importance of GxE interactions in human health.

Here we investigate the presence of GxE expression variants associated with long-term sun-exposure. Sun-exposure is associated with diverse human pathophysiologic phenotypes, from vitamin D synthesis to cancer [30–33]. As humans migrated across the world, changes in solar radiation exposure resulted in strong signatures of local adaptation [34]. Skin pigmentation genes have repeatedly arisen as some of the strongest examples of positive selection in humans [35–37]. Notable examples include *SLC45A2* and *SLC24A5*, which contain non-synonymous SNPs at high frequency in lighter-skin populations [34].

In addition to protein-coding changes, there is also evidence that non-coding changes have evolved in response to sun-exposure. Cis-regulatory regions of several skin-pigmentation genes show signs of selection, such as the intergenic regions of *KITLG* and the upstream enhancer of *OCA2* [34,38,39]. Polygenic signatures of selection have also been reported: eQTLs for genes down-regulated by UV exposure show significantly higher allele frequencies of the down-regulating allele in populations with higher UV exposure [40], suggesting that the dynamic response to UV may also be "hard-wired" via adaptive changes in eQTL allele frequencies. Limitations of these studies are that genetic control of gene expression and sun-exposure are often only indirectly linked (because the SNP, gene expression, and sun-exposure were not all measured in the same study) and they do not address whether the genetic control of gene expression itself may be dynamic and depend on the environment.

To identify genetic variants whose effect on gene expression depends on sun-exposure, we analyzed RNA-seq data from 607 skin samples [17] including sun-exposed skin (357 samples, hereafter denoted as SE) and non-sun-exposed skin (250 samples, hereafter denoted as NSE), which differ in lifetime sun-exposure. We then investigated whether the sun-exposure-dependent eQTLs show signs of local adaptation. Although evidence for positive selection of skin-pigmentation genes due to sun-exposure is strong, explanations for how these adaptations improved fitness are debated [33]. Hypotheses range across a gamut of health associations, including Vitamin D synthesis, folate-dependent neurological development, cancer, dehydration, and innate immunity [32,33]. By identifying GxE expression variants, we aim to identify the genes, and ultimately the specific traits, that have been critical for our recent adaptation to local climates around the world.

## Results

### The effect of sun-exposure on the skin transcriptome

**Fig 1:**
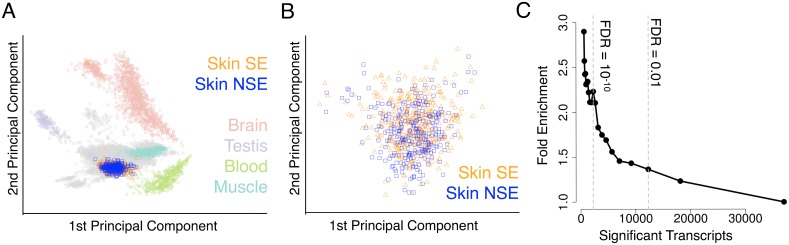
Sun-exposed and non-sun-exposed skin samples. (A) First and second principal components of all GTEx samples with selected tissue-types highlighted by color. (B) First and second principal components using all genes in only the skin samples. (C) Fold enrichment of 2-fold differentially expressed genes from Choi *et al*. [43] as a function of the stringency in calling differentially expressed genes from the GTEx samples.

We first examined the transcriptome-wide gene expression of the skin samples relative to the other tissues. Principal components of all GTEx samples were calculated. The 1st to 6th principal components primarily distinguished the samples by tissue, while the SE and NSE skin samples grouped together indistinguishably (Fig 1A, S1 Fig, Methods). Hierarchical clustering of the expression data also clustered the SE and the NSE samples together (S2 Fig, Methods). Thus, we estimate that sun-exposure has a subtle effect on gene expression in skin, when compared to the differences between tissues.

Because the principal component analysis was able to discriminate between tissues, we next investigated whether we could distinguish the exposure-type of the skin samples by performing the analysis on the skin samples alone. The first six principal components did not segregate the samples by exposure-type and the percent variance explained by each principal component was relatively small (PC1 explained 7.2% variance, PC2 explained 4.2%; percent variance cumulative in PC1-6 was 20.8%). This suggests that sun-exposure does not explain a large fraction of the variance in gene expression among these samples (Fig 1B, S3 Fig).

Despite this similarity in the SE and NSE samples, we have high power to detect differential expression because of the large sample sizes (196 NSE and 302 SE, after removal of technical replicates; see Methods). We performed differential expression analysis with DESeq2 [41], revealing 12,320 differentially expressed genes at a Benjamini-Hochberg FDR < 0.01 (out of 37,412 genes tested, S4 Fig, S1 Table). Among these genes, 522 genes had greater than 2-fold-change. We investigated the Gene Ontology enrichment of these genes using DAVID [42], identifying several enriched gene sets (S2 Table). Genes that were upregulated in SE were most strongly enriched for the GO term "Epithelial Cell Differentiation" (Bonferroni-corrected p = 1.5x10^-4^), whereas those upregulated in NSE were most strongly enriched for "Intermediate Filament" (Bonferroni-corrected p = 6.1x10^-9^).

As a critical control, we investigated whether the gene expression differences between the SE and NSE samples, which were obtained from the lower-leg and suprapubic skin of post-mortem samples, reflect effects of sun-exposure. To test this, we compared the list of differentially expressed genes with genes that were differentially expressed in human skin after repeated exposures to UVB [43]. We observed significant enrichment (Fisher's exact test p = 3.2x10^-6^ for differentially expressed genes at Benjamini-Hochberg FDR < 0.01, Fig 1C), indicating that sun-exposure differences in the post-mortem GTEx skin samples are broadly consistent with experimental UVB exposure. We also tested for overlap with genes differentially expressed in human skin fibroblasts in response to UV radiation [44], and found a similar trend (Fisher's exact test p = 1.6x10^-6^ for differentially expressed genes at Benjamini-Hochberg FDR < 0.01, S5 Fig). Investigating whether sun-exposure responses differed between ethnicities, we observed similar responses between individuals with predominantly European and African ancestry (see S1 Text).

### Differential cis-eQTLs: Sun-exposure association with the strength of cis-eQTL

**Fig 2:**
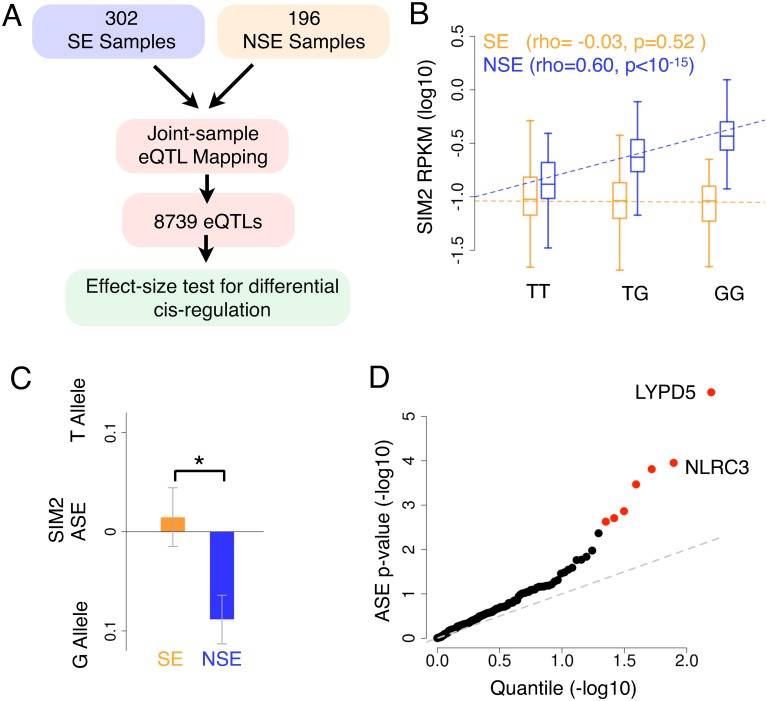
Differential cis-eQTL mapping and validation with ASE. (A) Outline of our approach to mapping differential cis-regulation. (B) The expression of *SIM2* for each exposure-type after segregation by genotype at the eQTL (rs2248813). P-values are from the asymptotic approximation of the Spearman correlation (rho). (C) ASE validation of the *SIM2* result for individuals heterozygous at the eQTL. The allele on the y-axis indicates the allele at the eQTL (see x-axis of panel B), and the ASE directionalities were phased based on these alleles. * indicates p-value < 0.05 by t-test. (D) QQ plot of each tested gene after combining the effect size test and the differential ASE test. Red indicates the gene had a significant GxE expression interaction with a SNP (FDR < 0.05, Benjamini-Hochberg).

We next investigated whether sun-exposure may affect cis-regulation, specifically at genomic loci that associate with mRNA levels (eQTLs). Previous studies of differential cis-regulation across tissues or environments have identified potential pitfalls that could lead to false positives, such as the “winner's curse” and differences in power, which we carefully aim to avoid in our analysis [45–47] (Fig 2A). To focus on the most replicable eQTLs, we first identified the strongest local eQTLs within 1 Mb of each gene. We used a Bayesian approach to map eQTLs in the SE samples and the NSE samples jointly (302 SE samples and 196 NSE samples after restricting to only samples with individual genotypes, mapped using eQTL-BMA [46]). By using only the strongest eQTL from joint-sample mapping, we remove the bias of inflated effect size estimates due to the “winner's curse”, a confounding effect that reduces the apparent replication rate when the strongest eQTL in one group is tested in another group [48]. eQTLs were selected as those that had a posterior probability greater than 0.95, resulting in 8739 eQTLs (1 SNP per gene, S6 Fig). These genes predominantly overlapped with GTEx eQTL target genes, and the posterior probabilities in our analysis were strongly correlated with the GTEx p-values, indicating that joint-sample mapping produces largely similar results (S7 Fig).

For each of the eQTLs, we then tested for significant differences in cis-eQTL effect between the SE and NSE samples. Unlike tests comparing eQTL significance between groups—which lead to false positives if low power in one group results in insignificant p-values—comparing the effect sizes (hereafter referred to as the effect-size test; [49]) does not result in false positives, since low power in one group would only decrease the ability to detect a significant difference in effect size. Applying this test to all 8739 eQTLs, we found 453 at a nominal p < 0.05, with only one eQTL significant at Benjamini-Hochberg FDR < 0.05. The most significant result was for *SIM2*, which was strongly affected by an eQTL in the NSE samples, but not in the SE samples (Fig 2B).

To validate these results, we analyzed allele specific expression (ASE) differences (differential ASE) between SE and NSE samples. ASE measures the effect of cis-regulation in individuals heterozygous for the regulatory variant. Thus, significant differential ASE between groups (comparing only the individuals who are heterozygous at the eQTL) indicates differential effects of the eQTL. Note that although ASE is measured using the same dataset, it is an independent source of validation because it examines the differences in expression within an individual rather than across individuals [23,25] (see simulation results in S8 Fig and S2 Text). As a control, we also confirmed that ASE identifies cis-regulatory effects in this dataset and is not biased between the SE and NSE groups (S9 Fig).

To test for validation of the *SIM2* sun-exposure-dependent eQTL (Fig 2B), we compared the ASE in all individuals who were heterozygous at the eQTL. Because the eQTL effect was present only in NSE samples, we predicted that the NSE heterozygotes would exhibit higher absolute magnitude of ASE than SE heterozygotes. In addition, because the G allele is associated with higher expression in the NSE eQTL analysis (Fig 2B), we predicted that the ASE of the NSE heterozygotes would be directed towards the G allele. We confirmed both predictions (Fig 2C), validating *SIM2* as the target of a GxE expression variant.

Extending this validation test to all genes, we found that results from differential ASE and the effect-size test are significantly concordant in directionality (S9 Fig). Concordance in directionality requires: 1) the same sample group (SE or NSE) must exhibit the stronger effect in both tests, and 2) the same allele must be the upregulating allele. The degree of concordance is likely underestimated by our analysis because the ASE data face several systematic limitations that reduce power, including imperfect phasing between the eQTL and SNPs inside the target transcript, and the small number of reads overlapping many heterozygous SNPs.

Because differential ASE provides independent evidence for a differential cis-regulatory effect, we combined both tests to identify additional genes with a significant effect. To combine the tests, we intersected significant SNPs by testing for differential ASE among genes with a nominally significant effect-size test (p < 0.05) and concordant ASE directionality (157 out of 289 genes concordant), resulting in seven significant differential eQTLs (Benjamini-Hochberg FDR < 0.05, Fig 2D). Four out of these seven genes show a stronger effect in the NSE samples, confirming that there is no evidence of bias due to differences in power between the groups (S3 Table). Only two of the seven genes exhibited significant differential expression at FDR < 0.05, and these two genes exhibited a stronger cis-eQTL effect in the environment with lower expression (S3 Table). This indicates that the trivial scenario of environment-specific gene expression silencing resulting in no evidence of cis-regulation for that particular environment is not occurring in these associations. Many of the genes have known roles in skin and sun exposure; for example *LYPD5*, the gene with the strongest effect, encodes the protein haldisin, which is associated with the late (outer) stages of skin differentiation [50,51]. *NLRC3*, the second strongest effect, is a gene that negatively regulates the *STING*-dependent innate immune pathway [52] that is activated after UV exposure [53,54].

### cis-reQTL mapping: Associating local SNPs with the effect of sun-exposure

**Fig 3:**
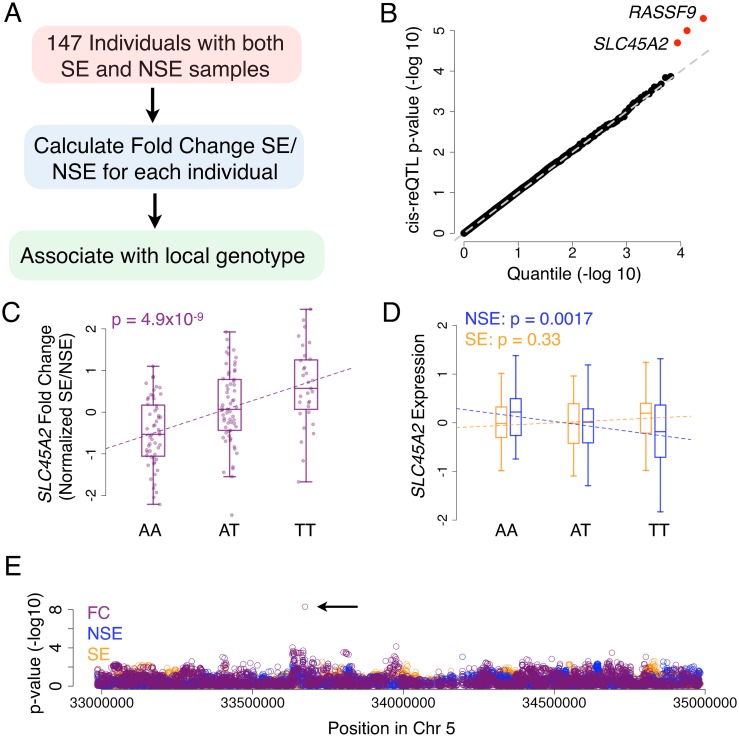
cis-reQTL mapping and *SLC45A2* reQTL. (A) Outline of the approach for mapping cis-reQTLs (B) QQ plot of the cis-reQTL p-value for all genes tested. Red indicates the top 3 genes discussed in the text and used in subsequent analyses. (C) Normalized fold change of *SLC45A2* expression separated by genotype at the cis-reQTL (rs12653176). p-value calculated from the Spearman correlation. (D) Normalized *SLC45A2* expression of the NSE and SE samples separated by genotype at the same locus. p-values calculated from the Spearman correlation. (E) Manhattan plot of the fold change (FC), NSE sample expression, and SE sample expression associations with all SNPs one megabase surrounding *SLC45A2*. p-values calculated from the Spearman correlation.

We then identified additional GxE expression variants by testing whether any local SNPs associate with the differential expression due to the sun-exposure of each individual (Fig 3A). This approach has been used in identifying GxE expression variants responding to immunological stimuli, referred to as cis-response eQTLs (cis-reQTLs) [23,26]. The advantage of this method is that comparing the expression within the same individual will account for the inter-individual differences, such as environmental variation, that can confound typical eQTL analysis. To map the cis-reQTLs, we first calculated the fold change difference in expression (SE / NSE) for all genes in the 147 individuals with both SE and NSE data (see Methods). We then tested the fold change values for association with SNPs within one megabase from the transcription start site of the gene, in a similar manner to eQTL mapping. As expected, there is a significant concordance between the cis-reQTL p-values and the eQTL effect-size test (S10 Fig). For the cis-reQTL analysis however, we also tested all local SNPs as opposed to just the strongest eQTLs, resulting in additional associations. The three most significant cis-reQTL genes were *RASSF9*, *NPIPL1*, and *SLC45A2* (Fig 3B; see Methods, p<2x10^-5^, Benjamini-Hochberg FDR<0.18). These cis-reQTLs were also nominally significant using the effect-size test (p = 5.0x10^-6^, p = 2.1x10^-5^, p = 2.7x10^-2^, respectively).

*SLC45A2* is a classic example of a skin-pigmentation gene under recent positive selection [37,55,56]. The gene encodes the Membrane-Associated Transporter Protein (MATP), containing a non-synonymous SNP (rs16891982) where the ancestral allele is nearly fixed in African and Asian populations while the derived allele is at high frequency with a north-south cline in European populations [34]. The cis-reQTL found in this study (rs12653176) is not in linkage disequilibrium with the previously-identified non-synonymous SNP in any tested populations (S11 Fig), and is located within the intron of the downstream gene *ADAMTS12*. The fold-change association in this cis-reQTL is more significant than either the SE or the NSE expression associations (Fig 3C and 3D), and *SLC45A2* had no significant cis-eQTL in skin (Fig 3E), suggesting that the cis-reQTL specifically controls the SE-response rather than baseline levels.

The strongest cis-reQTL in our analysis regulates *RASSF9*, a gene that is expressed highly in mouse epidermal keratinocytes [57]. *RASSF9* knockout mice were previously observed to have higher epidermal proliferation and abnormal differentiation, suggesting that the gene is involved in epidermal homeostasis.

### Evidence for local adaptation in sun-exposure-dependent regulatory SNPs

We then tested the differential cis-eQTLs and cis-reQTLs (hereafter referred to collectively as sun-exposure dependent eQTLs—se-eQTLs) for evidence of recent adaptation (S3 Table). Our hypothesis, based on previous findings [40,58], was that adaptation of human populations to local climates would lead to a correlation between the se-eQTL allele frequencies and the levels of solar radiation experienced by each population. To test this hypothesis, we used Bayenv 2.0, a Bayesian approach to test the significance of environmental associations while accounting for the relatedness between populations and the confidence in allele frequency estimates [59,60]. In a previous genome-wide analysis of 61 human populations, Bayenv identified evidence of local adaptation to solar radiation near several skin-related genes (e.g. *SLC45A2*, *KRT77*, and *OCA2*) [58].

**Fig 4:**
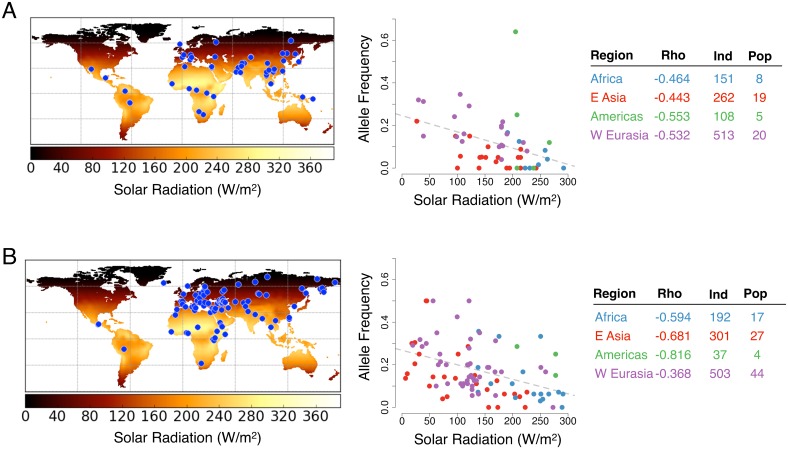
Testing se-eQTLs for signs of local adaptation. Environmental correlations with allele frequency in (A) HGDP populations and (B) Lazaridis et al [62] populations. Left panels: Heatmap of the environmental variables used in the study. Units are Watts per meter squared. Populations are marked with blue circles. Note that three of the American populations from Lazaridis *et al*. (Mixe, Mixtec, and Zapotec) [62] are marked in the same geographic location. Right panels: Radiation level vs allele frequency of the population for the *RASSF9* se-eQTL (rs11117173) with the color indicating the regional subgroup. For each regional subgroup, the Spearman correlation (Rho), the number of individuals (Ind), and the number of populations (Pop) are provided in the table.

Here we use Bayenv to test for association between solar radiation and the allele frequencies of the 10 se-eQTLs across the HGDP populations [60,61]. In the same manner as [58], we analyzed the winter and summer solar radiation separately. To estimate significance of the resulting Bayes factors, we calculated an empirical p-value using all skin eQTLs discovered in our study as background (see Methods). Among our 10 se-eQTLs, *RASSF9* had the strongest association with winter solar radiation (Fig 4A; empirical p = 6.1x10^-3^, S3 Table). Hancock *et al* [58] also found the strongest associations in skin pigmentation genes using the winter solar radiation as the environment, as opposed to summer solar radiation. We then validated the evidence for selection in *RASSF9* in another set of 92 populations while excluding HGDP individuals (empirical p = 0.040, Fig 4B, S3 Table, populations from [62]). The associations in both population sets were concordant across multiple continental regions (Spearman’s rho < -0.36, Fig 4), suggesting that this se-eQTL has been adaptive in multiple independent human lineages.

Commonly used haplotype-based tests for selection, such as EHH, did not show a significant increase for any particular se-eQTL, which may be an indication that the se-eQTLs are under very recent selection. As selective sweeps for large-effect skin-pigmentation genes have occurred within the last tens of thousands of years [37,63], we expect these small-effect events to also be recent as well. We tested this further using the singleton-density-score (SDS), a haplotype-based test of very recent selection performed on the UK10K Project data (Y. Field, N. Telis, and J. Pritchard, pers. comm., [64]). The se-eQTL for *RASSF9* showed the strongest evidence of selection (Bonferroni corrected p = 0.018), lending further support to the hypothesis of recent selection acting on this locus.

## Discussion

Our study of sun-exposure dependent regulation of gene expression identified several examples of GxE interactions where genetic variants affect the gene expression response to sun exposure. In contrast to our differential expression analysis that identified over 12,000 transcripts—an unwieldy number that makes it challenging to uncover the causal or independent effects—focusing on GxE interactions allowed us to identify 10 independent loci directly associated with the sun-exposure response, with the strongest hits having known skin or sun-exposure related functions. This small number of significant GxE expression variants is consistent with previous observations that the number of GxE effects detected is smaller than additive effects [25].

We identified GxE expression variants using two methods, differential cis-eQTL analysis and cis-reQTLs. Although both methods uncover GxE expression variants, we found that each provided unique results in their top associations. The two methods have distinct advantages: differential cis-eQTL analysis utilized information from a larger number of samples because there was no requirement for individuals to have both the SE and NSE data, while the cis-reQTL analysis controlled for possible individual-level confounding factors (such as genetic background or environmental variation) because it compared the SE and NSE samples within the same individuals. In addition, our differential cis-eQTL analysis was based on eQTLs identified via joint-sample mapping in order to reduce the effect of the winner's curse. As a result, the differential cis-eQTL analysis preferentially analyzed SNPs with eQTL effects in both tissues, while the cis-reQTL approach agnostically considered all local SNPs. Despite these differences, overall the methods were generally concordant (S10 Fig).

Building on previous studies, we also found that allele-specific expression is an effective, orthogonal approach to identify GxE expression variants [25]. Combining the differential ASE test with the effect-size test strengthened the significance of our findings; therefore we recommend investigating both allele-specific and non-allele-specific expression whenever possible.

An important control in our analysis was confirming that the differences in gene expression of the SE and NSE samples reflected differences in sun-exposure. Because the samples were obtained from the post-mortem lower-leg and suprapubic skin, respectively, it is important to justify that the downstream analyses reflect differences caused by sun-exposure as opposed to differences unrelated to sun exposure. The relevance of sun-exposure was validated by significant concordance of the differentially expressed genes with two independent experimental approaches, one with whole skin UV-B treatment and another with skin fibroblast UV exposure. In addition, the significance of *SLC45A2*, a gene previously determined to be under selection due to solar radiation, also suggests that sun-exposure is likely driving this effect. Lastly, *SLC45A2* and *RASSF9* have both been found to be differentially expressed in response to UV-B treatment [43], thus indicating that sun-exposure is likely affecting these genes' expression.

In addition to sample sizes, statistical power can also affect GxE interaction studies via a large multiple-hypothesis testing burden. This limitation was a key factor in our choice of testing only local GxE expression variants and refraining from testing for distant trans-associations. Local eQTLs tend to be highly replicable with strong effect sizes and finding them requires testing only a small number of SNPs per gene. In contrast, trans-eQTLs are challenging to map if every SNP is tested for each gene, though it is possible to identify trans-acting GxE expression variants in well-controlled studies [18,20,29]. Larger sample sizes or testing only SNPs with a high prior probability of participating in trans-GxE effects can also boost power to map them.

Overall, the GxE expression variants did not exhibit the strong population-specific fixation patterns observed in classical examples of positive selection. This however does not necessarily indicate that GxE expression variants are in general weakly selected. Mapping GxE interactions requires variation not only in environment but also in genotype. Because strong selection will remove genetic variation, our GxE mapping approach may not detect associations with the most strongly selected SNPs. Because the GxE mapping process focuses on polymorphic variants, we may detect signs of selection that are perhaps more recent or complex.

Although the evidence for human evolution in response to sun-exposure changes is strong, hypotheses for how adaptations in skin-pigmentation genes have led to increased fitness are debated [32,33]. To our knowledge, the se-eQTL genes, aside from *SLC45A2*, were not previously associated with human adaptation to sun-exposure. We found evidence that the se-eQTL of *RASSF9* has been subject to local adaptation using two independent population cohorts as well as from a test for very recent adaptation in Europeans. Our finding of adaptation involving *RASSF9*, a gene involved in epidermal homeostasis and differentiation, supports the idea that skin pigmentation is not the only trait shaped by adaptation to sun-exposure [65,66].

The approaches we employed could be applied to investigate GxE interactions involving any pair of tissues; although environment is typically considered to be extrinsic to an organism, cell type can be considered a major determinant of each gene’s intracellular environment. As more GxE interactions are discovered, we can then study their relationships with diseases or other traits to better understand their role in phenotypic variation and adaptation.

## Methods

### Data

Expression and genotype data for sun-exposed (SE) and non-sun-exposed (NSE) skin samples were obtained from GTEx v6 [17]. Genotype data are from the HumanOmni5-4v1_B and the HumanOmni2.5-8v1-1_B platform with variants imputed using IMPUTE2 and the 1000 Genome Project Phase 1 version 3 reference panel (genotyping and imputation performed by GTEx consortium). To account for platform bias, we included genotyping platform as covariates in downstream analyses as described below. Sun-exposed skin samples were taken from the lower leg, and the non-sun-exposed skin samples were taken from the suprapubic area. As described in the GTEx resources, the skin samples were obtained as slices with the subcutaneous fat trimmed off, avoiding pubic hair in the suprapubic region, and subsequently fixed and stored in PaxGene tissue container. For the genotype data, we used all SNPs that passed the filters in the phased/imputed dataset (INFO > 0.4, Hardy Weinberg p-value > 10^−6^, call-rate > 95%). For the expression data, we used the samples selected by GTEx as adequate for eQTL analysis (which were selected based on quality). Gene models for all expression analyses were also obtained from GTEx, which combined the transcripts annotated by GENCODE v19 for each gene. Principal components in the all-sample analysis were calculated from the RPKM expression of 1000 randomly-sampled genes with a mean RPKM > 1. Random sampling was performed to reduce computation time—repeating the sampling produced similar results. Hierarchical clustering was performed with the Euclidean distance between tissues using the tissue-specific mean expression across all genes. Principal components of the skin-only analysis were obtained using all genes with mean RPKM > 1 across the skin samples.

### Differential Expression Analyses

We investigated differential expression between the sun-exposed and non-sun-exposed skin samples using the raw read counts obtained from the GTEx consortium (which used RNASeQCv1.1.8 to estimate values). Genes with zero counts across all samples were removed. Differential expression was calculated using the DESeq function in the DESeq2 package [41] using default parameters. Gene Ontology enrichment was assessed using the Ensemble Gene IDs with DAVID [42]. All genes that were tested for differential expression were used as the gene background and the Gene Ontology BP, CC, and MF collections were tested. For the ancestry-specific differential expression analysis, genotyped individuals were separated into European ancestry (377 individuals) and African ancestry (68 individuals) as determined by principal component analyses. Europeans ancestry was designated as individuals with PC1 <-0.01 and PC2 > -0.04) and African ancestry population was designated as individuals with PC1 > 0.1. This classification was concordant with the individuals' reported race (S12 Fig). We then tested each gene for evidence of ancestry-specific sun-exposure differential expression by assessing the significance of the ethnicity-environment interaction term in a linear model. The likelihood ratio test was performed within DESeq2, comparing the models (Expression ~ ancestry + exposure, and Expression ~ ancestry + exposure + ancestry:exposure).

### Joint sample eQTL mapping

Joint sample eQTL mapping was performed using eQTL-BMA with both SE and NSE samples [46]. To maximize the number of samples and to maintain consistency with the GTEx eQTL association mapping, we used all individuals (including individuals of varying ancestry) in the analysis. To control for effects of ancestry and other confounding factors, we used all covariates used by GTEx in their eQTL association mapping, which includes the first three genotype principal components, PEER factors, genotyping array platform, and gender. For input of the expression data, we used the residuals after regressing out covariates from the normalized expression matrix provided by GTEx (normalization used by the GTEx: RPKM data, low-expression filtered, quantile normalized, fitted to standard normal). eQTLs were mapped to SNPs within 10^6^ bp of the TSS, with the parameters "—bfs all—error hybrid—maf 0.05—qnorm—analys join" and using the large and small grid provided by the package. This window size of 10^6^ bp was selected to be consistent with the GTEx Consortium analysis [17]. The configuration weights were determined using the hierarchical model and pi0 was calculated using the EBF procedure as specified by the package.

### Allele-specific expression calculation

For the allele-specific expression analysis, reads provided by GTEx were mapped using STAR (—outFilterMultimapNmax 1,—clip 5pNbases 6) [67] to the GRCh37.p13 human genome masked with all SNPs in dbSNP142. Duplicate reads were removed using a combination of in-house scripts and Samtools Rmdup to remove duplicate reads randomly (instead of selecting the read with the highest quality score) as suggested previously [68,69]. Allele-specific expression was calculated at each exonic SNP, with a random SNP chosen when a read overlapped multiple sites. Mono-allelic sites were removed as recommended [69] to reduce bias and the gene-level exon counts were summed using the GTEx phased SNP calls. Using these counts, the value of ASE for each gene-individual pair was calculated as allele1 / (allele1 + allele2), requiring at least 3 reads. For comparison between ASE results and differential eQTL analysis, we required at least 5 ASE measurements for SE homozygotes, SE heterozygotes, NSE homozygotes, and NSE heterozygotes.

### Differential cis-eQTL analysis by effect-size test

The effect-size test was performed as described in Fraser *et al*. [49]. Pearson correlation for SE and NSE samples were calculated using the normalized expression matrices used by GTEx eQTL analysis. Each correlation coefficient was converted to a z-score by 0.5*log((1+r)/(1-r)). The difference in effect size was then tested by calculating the value t = (z_SE_—z_NSE_) / sqrt (1/(n_SE_ -3) + 1/ (n_NSE_-3)), which is normally distributed. z_SE_ and z_NSE_ denote the z-scores for the SE and NSE samples. n_SE_ and n_NSE_ denote the number of samples used in the correlation coefficient calculation.

### cis-reQTL analysis

cis-reQTL analysis was performed using the subset of individuals analyzed in the differential eQTL analysis who had both SE and NSE data. To calculate fold change, the RPKM values for each sample were first quantile normalized across samples. Fold change (SE / NSE) was then calculated for each gene-individual pair, with values of 0 set to the minimum non-zero value of that gene. The log fold change values for each gene were then fit to a standard normal. Hidden covariates were then discovered using PEER (k = 15) [70]. In order to correct for hidden covariates most relevant to these log-ratios, PEER covariates were assessed for these directly (as opposed to using RPKM values). The first 15 PEER covariates, gender, sequencing platform, and the first 3 genotype principal components were used as covariates for mapping (Results remained consistent after also correcting for age). Mapping was performed with the linear model of Matrix eQTL using SNPs with MAF > 0.05 found within 10^6^ bp from the transcription start site [71]. The window size of 10^6^ bp was selected to remain consistent with the GTEx Consortium analysis for cis-eQTL mapping [17]. Significance of the SNP with the strongest association for each gene was assessed by comparing the nominal p-value with the p-values of the best-associating-SNPs from at least 10^3^ permutations of the individual labels.

### Testing for selection using Bayenv

Local adaptation was tested using Bayenv2.0 using the Human Genome Diversity Panel (HGDP) and HapMap samples [60,61] and an independent set of 92 populations [62]. The Lazaridis et al. data [62] were filtered to remove African American individuals, HGDP individuals, and populations with fewer than five individuals. The population covariance matrix was estimated with 10,000 random SNPs selected from the genotyped SNPs with linked SNPs removed by PLINK v1.9 (—indep-pairwise 1000kb 1 0.2) and k = 1000. Many of the se-eQTLs were not genotyped by HGDP or Lazaridis et al., and thus the allele frequencies were estimated by first imputing the genotypes for each individual with IMPUTE 2 [72] using the 1000 Genomes Phase 3 reference panel and default parameters. Bayenv results have previously been observed to be unstable with a small number of iterations [73], so we took the median of 10 runs with k = 500,000. To estimate the significance of the Bayes factors, we performed the same analyses on all eQTLs discovered in our analysis to use as the background.

Climate data were obtained from the NCEP/NCAR Reanalysis [74] using the monthly long-term mean of the downward short-wave radiation flux to match the environmental variables used by Hancock *et al*. [58]. The summer radiation was calculated by averaging the months of June, July, August for positive latitudes and December, January, February for negative latitudes. The winter radiation was calculated by averaging the opposite months. Values were then standardized for Bayenv analysis.

## Supporting Information

S1 Text Comparison of sun-exposure differential expression between ancestries. (PDF)

S2 Text Simulation to demonstrate independence of effect-size test and differential ASE. (PDF)

S1 Fig 3rd-6th principal components of expression from all GTEx samples with select tissue of origin highlighted. (PDF)

S2 Fig Hierarchical clustering of all tissue-types using the Euclidean distance of the tissue-specific mean gene expression. (PDF)

S3 Fig 3rd-6th principal components of the expression from skin samples. (PDF)

S4 Fig Volcano plot of differential expression. (PDF)

S5 Fig Fold enrichment of differentially expressed genes from Gentile *et al*. [44] as a function of the stringency in calling differentially expressed genes from the GTEx samples. (PDF)

S6 Fig Posterior probability of the gene having an eQTL from joint-sample eQTL mapping of the SE and NSE samples by eqtlBMA. 8739 genes have > 0.95 posterior probability marked by the red dotted line.
(PDF)

S7 Fig (A) Skin eQTLs from our BMA analysis overlap with skin eQTLs from GTEx. (B) GTEx -log10 P-values of the eQTL correlate significantly with the posterior probability from in BMA. Only eQTLs found in the GTEx analysis were used in this correlation.
(PDF)

S8 Fig (A) 1000 simulations of an exposure-specific eQTL indicate that the differential ASE test and effect-size test have significantly correlated results. This indicates that both methods can be used to detect the signal of differential cis-regulation. (B) 1000 simulations of the null (absence of an exposure-specific eQTL) indicate that the effect-size test and the differential ASE analysis do not show a correlated result, even though both tests use data derived from the same simulation. This indicates that the differential ASE analysis and the effect-size test are independent tests. See S2 Text for details on simulations.
(PDF)

S9 Fig (A) Absolute ASE of all cis-eQTLs, separated by measurements when the individual was homozygous (Hom) at the eQTL or heterozygous at the eQTL (Het). Significance was assessed by Wilcoxon rank-sum test. *** marks p < 10^−15^. Note that the magnitude of the effect size is similar to other ASE analyses [69] (B) Absolute ASE of all cis-eQTLs separated by genotype at the eQTL and exposure-type. Significance was assessed by Wilcoxon rank-sum test. NS marks the p >0.05. *** marks p < 10^−15^. (C) Significance of concordant directionality between the effect-size test and the differential ASE analysis as a function of the p-value cutoff in the differential ASE analysis. Directionality p-value is calculated by the two-sided binomial test, where the expected fraction of concordance is set at the concordance of all genes (0.36).
(PDF)

S10 Fig (A) Effect-size p-value vs cis-reQTL p-value for all associations that were tested in both analyses (1 per gene). P-value is calculated from the Spearman correlation.
(PDF)

S11 Fig *SLC45A2* cis-reQTL is unlinked to known selected loci. The cis-reQTL found in this study (rs12653176) is located at the bottom and the known selected non-synonymous SNP (rs16891982) is located at the top. The distance between the two SNPs is 278kb.
(PDF)

S12 Fig (A) Principal components of genotype (calculated by GTEx) separate individuals into ancestry groups. Red is used to mark the reported race “White”. Blue marks the reported race of “Black or African American”. Empty circles represent other reported race. Red shaded region demarcate the designated principal component boundaries in this study for the European ancestry population resulting in 377 genotyped individuals. Blue shaded region demarcates the African ancestry population, resulting in 68 genotyped individuals. (B) The fold change, log (SE / NSE), of all genes in the European ancestry individuals and the African ancestry individuals. P-value was calculated based on the asymptotic approximation of the Spearman correlation (rho).
(PDF)

S13 Fig Expression of *TCN1* separated by the SE and NSE samples with European and African ancestry. Even with the substantially lower power in the African ancestry individuals, we see a significant difference in differential expression in this gene at a Benjamini-Hochberg FDR < 0.01 (p = 5.6x10^-7^, likelihood ratio test).
(PDF)

S1 Table Differential expression results between SE and NSE samples. (XLS)

S2 Table Gene Ontology enrichment for genes that are differentially expressed (Benjamini-Hochberg FDR < 0.01) with greater than two-fold change. (XLS)

S3 Table List of se-eQTLs tested for selection and the selection scores/p-values. The higher expressed exposure-type is indicated when significant (Benjamini-Hochberg FDR<0.05). The exposure-type with the stronger effect according to the effect-size test is indicated, and the analysis from where the se-eQTL was obtained is also indicated.
(XLS)

## References

[1] Lynch M, Walsh B (1998) Genetics and analysis of quantitative traits. Sunderland, Mass.: Sinauer xvi, 980 p. p.

[2] Khoury MJ, Adams MJ Jr., Flanders WD (1988) An epidemiologic approach to ecogenetics. Am J Hum Genet 42: 89–95. 3337114 PMC1715339

[3] Koch R, de la Cruz F (1999) Historical aspects and overview of research on phenylketonuria. Mental Retardation and Developmental Disabilities Research Reviews 5: 101–103. 10.1002/(SICI)1098-2779(1999)5:2%3C101::AID-MRDD1%3E3.0.CO;2-D

[4] Roses AD (2004) Pharmacogenetics and drug development: the path to safer and more effective drugs. Nat Rev Genet 5: 645–656. 10.1038/nrg1432 15372086

[5] Weinshilboum RM, Wang L (2006) Pharmacogenetics and pharmacogenomics: development, science, and translation. Annu Rev Genomics Hum Genet 7: 223–245. 10.1146/annurev.genom.6.080604.162315 16948615

[6] Via S, Gomulkiewicz R, De Jong G, Scheiner SM, Schlichting CD, et al (1995) Adaptive phenotypic plasticity: consensus and controversy. Trends Ecol Evol 10: 212–217. 10.1016/S0169-5347(00)89061-8 21237012

[7] Eichler EE, Flint J, Gibson G, Kong A, Leal SM, et al (2010) Missing heritability and strategies for finding the underlying causes of complex disease. Nat Rev Genet 11: 446–450. 10.1038/nrg2809 20479774 PMC2942068

[8] Stratton DA (1994) Genotype-by-Environment Interactions for Fitness of Erigeron annuus Show Fine-Scale Selective Heterogeneity. Evolution 48: 1607 10.2307/2410251 28568414

[9] Manolio TA, Collins FS, Cox NJ, Goldstein DB, Hindorff LA, et al (2009) Finding the missing heritability of complex diseases. Nature 461: 747–753. 10.1038/nature08494 19812666 PMC2831613

[10] Zuk O, Hechter E, Sunyaev SR, Lander ES (2012) The mystery of missing heritability: Genetic interactions create phantom heritability. Proc Natl Acad Sci U S A 109: 1193–1198. 10.1073/pnas.1119675109 22223662 PMC3268279

[11] Hunter DJ (2005) Gene-environment interactions in human diseases. Nat Rev Genet 6: 287–298. 10.1038/nrg1578 15803198

[12] Flint J, Mackay TF (2009) Genetic architecture of quantitative traits in mice, flies, and humans. Genome Res 19: 723–733. 10.1101/gr.086660.108 19411597 PMC3647534

[13] Grishkevich V, Yanai I (2013) The genomic determinants of genotype x environment interactions in gene expression. Trends Genet 29: 479–487. 10.1016/j.tig.2013.05.006 23769209

[14] van Nas A, Ingram-Drake L, Sinsheimer JS, Wang SS, Schadt EE, et al (2010) Expression quantitative trait loci: replication, tissue- and sex-specificity in mice. Genetics 185: 1059–1068. 10.1534/genetics.110.116087 20439777 PMC2907192

[15] Innocenti F, Cooper GM, Stanaway IB, Gamazon ER, Smith JD, et al (2011) Identification, replication, and functional fine-mapping of expression quantitative trait loci in primary human liver tissue. PLoS Genet 7: e1002078 10.1371/journal.pgen.1002078 21637794 PMC3102751

[16] Powell JE, Henders AK, McRae AF, Kim J, Hemani G, et al (2013) Congruence of additive and non-additive effects on gene expression estimated from pedigree and SNP data. PLoS Genet 9: e1003502 10.1371/journal.pgen.1003502 23696747 PMC3656157

[17] Consortium GT (2015) Human genomics. The Genotype-Tissue Expression (GTEx) pilot analysis: multitissue gene regulation in humans. Science 348: 648–660. 10.1126/science.1262110 25954001 PMC4547484

[18] Li Y, Alvarez OA, Gutteling EW, Tijsterman M, Fu J, et al (2006) Mapping determinants of gene expression plasticity by genetical genomics in C. elegans. PLoS Genet 2: e222 10.1371/journal.pgen.0020222 17196041 PMC1756913

[19] Landry CR, Oh J, Hartl DL, Cavalieri D (2006) Genome-wide scan reveals that genetic variation for transcriptional plasticity in yeast is biased towards multi-copy and dispensable genes. Gene 366: 343–351. 10.1016/j.gene.2005.10.042 16427747

[20] Smith EN, Kruglyak L (2008) Gene-environment interaction in yeast gene expression. PLoS Biol 6: e83 10.1371/journal.pbio.0060083 18416601 PMC2292755

[21] Gat-Viks I, Chevrier N, Wilentzik R, Eisenhaure T, Raychowdhury R, et al (2013) Deciphering molecular circuits from genetic variation underlying transcriptional responsiveness to stimuli. Nat Biotechnol 31: 342–349. 10.1038/nbt.2519 23503680 PMC3622156

[22] Hemani G, Shakhbazov K, Westra HJ, Esko T, Henders AK, et al (2014) Detection and replication of epistasis influencing transcription in humans. Nature 508: 249–253. 10.1038/nature13005 24572353 PMC3984375

[23] Lee MN, Ye C, Villani AC, Raj T, Li W, et al (2014) Common genetic variants modulate pathogen-sensing responses in human dendritic cells. Science 343: 1246980 10.1126/science.1246980 24604203 PMC4124741

[24] Fairfax BP, Humburg P, Makino S, Naranbhai V, Wong D, et al (2014) Innate immune activity conditions the effect of regulatory variants upon monocyte gene expression. Science 343: 1246949 10.1126/science.1246949 24604202 PMC4064786

[25] Knowles DA, Davis JR, Raj A, Zhu X, Potash JB, et al (2015) Allele-specific expression reveals interactions between genetic variation and environment. bioRxiv. 10.1038/nmeth.4298 PMC5501199 28530654

[26] Caliskan M, Baker SW, Gilad Y, Ober C (2015) Host genetic variation influences gene expression response to rhinovirus infection. PLoS Genet 11: e1005111 10.1371/journal.pgen.1005111 25874939 PMC4395341

[27] Barreiro LB, Tailleux L, Pai AA, Gicquel B, Marioni JC, et al (2012) Deciphering the genetic architecture of variation in the immune response to Mycobacterium tuberculosis infection. Proc Natl Acad Sci U S A 109: 1204–1209. 10.1073/pnas.1115761109 22233810 PMC3268270

[28] Mangravite LM, Engelhardt BE, Medina MW, Smith JD, Brown CD, et al (2013) A statin-dependent QTL for GATM expression is associated with statin-induced myopathy. Nature 502: 377–380. 10.1038/nature12508 23995691 PMC3933266

[29] Smirnov DA, Morley M, Shin E, Spielman RS, Cheung VG (2009) Genetic analysis of radiation-induced changes in human gene expression. Nature 459: 587–591. 10.1038/nature07940 19349959 PMC3005325

[30] Gillie O (2006) A new government policy is needed for sunlight and vitamin D. Br J Dermatol 154: 1052–1061. 10.1111/j.1365-2133.2006.07261.x 16704634

[31] Lautenschlager S, Wulf HC, Pittelkow MR (2007) Photoprotection. Lancet 370: 528–537. 10.1016/S0140-6736(07)60638-2 17693182

[32] Moan J, Porojnicu AC, Dahlback A, Setlow RB (2008) Addressing the health benefits and risks, involving vitamin D or skin cancer, of increased sun exposure. Proc Natl Acad Sci U S A 105: 668–673. 10.1073/pnas.0710615105 18180454 PMC2206594

[33] Jablonski NG, Chaplin G (2010) Colloquium paper: human skin pigmentation as an adaptation to UV radiation. Proc Natl Acad Sci U S A 107 Suppl 2: 8962–8968. 10.1073/pnas.0914628107 20445093 PMC3024016

[34] Sturm RA, Duffy DL (2012) Human pigmentation genes under environmental selection. Genome Biol 13: 248 10.1186/gb-2012-13-9-248 23110848 PMC3491390

[35] Pickrell JK, Coop G, Novembre J, Kudaravalli S, Li JZ, et al (2009) Signals of recent positive selection in a worldwide sample of human populations. Genome Res 19: 826–837. 10.1101/gr.087577.108 19307593 PMC2675971

[36] Grossman SR, Shlyakhter I, Karlsson EK, Byrne EH, Morales S, et al (2010) A composite of multiple signals distinguishes causal variants in regions of positive selection. Science 327: 883–886. 10.1126/science.1183863 20056855

[37] Mathieson I, Lazaridis I, Rohland N, Mallick S, Patterson N, et al (2015) Genome-wide patterns of selection in 230 ancient Eurasians. Nature 528: 499–503. 10.1038/nature16152 26595274 PMC4918750

[38] Miller CT, Beleza S, Pollen AA, Schluter D, Kittles RA, et al (2007) cis-Regulatory changes in Kit ligand expression and parallel evolution of pigmentation in sticklebacks and humans. Cell 131: 1179–1189. 10.1016/j.cell.2007.10.055 18083106 PMC2900316

[39] Visser M, Kayser M, Palstra RJ (2012) HERC2 rs12913832 modulates human pigmentation by attenuating chromatin-loop formation between a long-range enhancer and the OCA2 promoter. Genome Res 22: 446–455. 10.1101/gr.128652.111 22234890 PMC3290780

[40] Fraser HB (2013) Gene expression drives local adaptation in humans. Genome Res 23: 1089–1096. 10.1101/gr.152710.112 23539138 PMC3698502

[41] Love MI, Huber W, Anders S (2014) Moderated estimation of fold change and dispersion for RNA-seq data with DESeq2. Genome Biol 15: 550 10.1186/s13059-014-0550-8 25516281 PMC4302049

[42] Huang da W, Sherman BT, Lempicki RA (2009) Systematic and integrative analysis of large gene lists using DAVID bioinformatics resources. Nat Protoc 4: 44–57. 10.1038/nprot.2008.211 19131956

[43] Choi W, Miyamura Y, Wolber R, Smuda C, Reinhold W, et al (2010) Regulation of human skin pigmentation in situ by repetitive UV exposure: molecular characterization of responses to UVA and/or UVB. J Invest Dermatol 130: 1685–1696. 10.1038/jid.2010.5 20147966 PMC3478754

[44] Gentile M, Latonen L, Laiho M (2003) Cell cycle arrest and apoptosis provoked by UV radiation-induced DNA damage are transcriptionally highly divergent responses. Nucleic Acids Res 31: 4779–4790. 10.1093/nar/gkg675 12907719 PMC169943

[45] Dimas AS, Deutsch S, Stranger BE, Montgomery SB, Borel C, et al (2009) Common regulatory variation impacts gene expression in a cell type-dependent manner. Science 325: 1246–1250. 10.1126/science.1174148 19644074 PMC2867218

[46] Flutre T, Wen X, Pritchard J, Stephens M (2013) A Statistical Framework for Joint eQTL Analysis in Multiple Tissues. PLoS Genet 9: e1003486 10.1371/journal.pgen.1003486 23671422 PMC3649995

[47] Gutierrez-Arcelus M, Ongen H, Lappalainen T, Montgomery SB, Buil A, et al (2015) Tissue-specific effects of genetic and epigenetic variation on gene regulation and splicing. PLoS Genet 11: e1004958 10.1371/journal.pgen.1004958 25634236 PMC4310612

[48] Zollner S, Pritchard JK (2007) Overcoming the winner's curse: estimating penetrance parameters from case-control data. Am J Hum Genet 80: 605–615. 10.1086/512821 17357068 PMC1852705

[49] Fraser HB, Lam LL, Neumann SM, Kobor MS (2012) Population-specificity of human DNA methylation. Genome Biol 13: R8 10.1186/gb-2012-13-2-r8 22322129 PMC3334571

[50] Kriegbaum MC, Clausen OP, Laerum OD, Ploug M (2015) Expression of the Ly6/uPAR-domain proteins C4.4A and Haldisin in non-invasive and invasive skin lesions. J Histochem Cytochem 63: 142–154. 10.1369/0022155414563107 25414274 PMC4305517

[51] Gardsvoll H, Kriegbaum MC, Hertz EP, Alpizar-Alpizar W, Ploug M (2013) The urokinase receptor homolog Haldisin is a novel differentiation marker of stratum granulosum in squamous epithelia. J Histochem Cytochem 61: 802–813. 10.1369/0022155413501879 23896969 PMC3808577

[52] Zhang L, Mo J, Swanson KV, Wen H, Petrucelli A, et al (2014) NLRC3, a member of the NLR family of proteins, is a negative regulator of innate immune signaling induced by the DNA sensor STING. Immunity 40: 329–341. 10.1016/j.immuni.2014.01.010 24560620 PMC4011014

[53] Gallo RL, Bernard JJ (2014) Innate immune sensors stimulate inflammatory and immunosuppressive responses to UVB radiation. J Invest Dermatol 134: 1508–1511. 10.1038/jid.2014.32 24825061 PMC4271625

[54] Kemp MG, Lindsey-Boltz LA, Sancar A (2015) UV Light Potentiates STING (Stimulator of Interferon Genes)-dependent Innate Immune Signaling through Deregulation of ULK1 (Unc51-like Kinase 1). J Biol Chem 290: 12184–12194. 10.1074/jbc.M115.649301 25792739 PMC4424351

[55] Soejima M, Tachida H, Ishida T, Sano A, Koda Y (2006) Evidence for recent positive selection at the human AIM1 locus in a European population. Mol Biol Evol 23: 179–188. 10.1093/molbev/msj018 16162863

[56] Bin BH, Bhin J, Yang SH, Shin M, Nam YJ, et al (2015) Membrane-Associated Transporter Protein (MATP) Regulates Melanosomal pH and Influences Tyrosinase Activity. PLoS One 10: e0129273 10.1371/journal.pone.0129273 26057890 PMC4461305

[57] Lee CM, Yang P, Chen LC, Chen CC, Wu SC, et al (2011) A novel role of RASSF9 in maintaining epidermal homeostasis. PLoS One 6: e17867 10.1371/journal.pone.0017867 21445300 PMC3061870

[58] Hancock AM, Witonsky DB, Alkorta-Aranburu G, Beall CM, Gebremedhin A, et al (2011) Adaptations to climate-mediated selective pressures in humans. PLoS Genet 7: e1001375 10.1371/journal.pgen.1001375 21533023 PMC3080864

[59] Coop G, Witonsky D, Di Rienzo A, Pritchard JK (2010) Using environmental correlations to identify loci underlying local adaptation. Genetics 185: 1411–1423. 10.1534/genetics.110.114819 20516501 PMC2927766

[60] Gunther T, Coop G (2013) Robust identification of local adaptation from allele frequencies. Genetics 195: 205–220. 10.1534/genetics.113.152462 23821598 PMC3761302

[61] Li JZ, Absher DM, Tang H, Southwick AM, Casto AM, et al (2008) Worldwide human relationships inferred from genome-wide patterns of variation. Science 319: 1100–1104. 10.1126/science.1153717 18292342

[62] Lazaridis I, Patterson N, Mittnik A, Renaud G, Mallick S, et al (2014) Ancient human genomes suggest three ancestral populations for present-day Europeans. Nature 513: 409–413. 10.1038/nature13673 25230663 PMC4170574

[63] Beleza S, Santos AM, McEvoy B, Alves I, Martinho C, et al (2013) The timing of pigmentation lightening in Europeans. Mol Biol Evol 30: 24–35. 10.1093/molbev/mss207 22923467 PMC3525146

[64] Field Y, Boyle EA, Telis N, Gao Z, Gaulton KJ, et al (2016) Detection of human adaptation during the past 2,000 years. bioRxiv. 10.1126/science.aag0776 PMC5182071 27738015

[65] Gautam P, Chaurasia A, Bhattacharya A, Grover R, Indian Genome Variation C, et al (2015) Population diversity and adaptive evolution in keratinization genes: impact of environment in shaping skin phenotypes. Mol Biol Evol 32: 555–573. 10.1093/molbev/msu342 25534032

[66] Marques PI, Fonseca F, Sousa T, Santos P, Camilo V, et al (2016) Adaptive Evolution Favoring KLK4 Downregulation in East Asians. Mol Biol Evol 33: 93–108. 10.1093/molbev/msv199 26420451

[67] Dobin A, Davis CA, Schlesinger F, Drenkow J, Zaleski C, et al (2013) STAR: ultrafast universal RNA-seq aligner. Bioinformatics 29: 15–21. 10.1093/bioinformatics/bts635 23104886 PMC3530905

[68] van de Geijn B, McVicker G, Gilad Y, Pritchard JK (2015) WASP: allele-specific software for robust molecular quantitative trait locus discovery. Nat Methods 12: 1061–1063. 10.1038/nmeth.3582 26366987 PMC4626402

[69] Castel SE, Levy-Moonshine A, Mohammadi P, Banks E, Lappalainen T (2015) Tools and best practices for data processing in allelic expression analysis. Genome Biol 16: 195 10.1186/s13059-015-0762-6 26381377 PMC4574606

[70] Stegle O, Parts L, Durbin R, Winn J (2010) A Bayesian framework to account for complex non-genetic factors in gene expression levels greatly increases power in eQTL studies. PLoS Comput Biol 6: e1000770 10.1371/journal.pcbi.1000770 20463871 PMC2865505

[71] Shabalin AA (2012) Matrix eQTL: ultra fast eQTL analysis via large matrix operations. Bioinformatics 28: 1353–1358. 10.1093/bioinformatics/bts163 22492648 PMC3348564

[72] Howie BN, Donnelly P, Marchini J (2009) A flexible and accurate genotype imputation method for the next generation of genome-wide association studies. PLoS Genet 5: e1000529 10.1371/journal.pgen.1000529 19543373 PMC2689936

[73] Blair LM, Granka JM, Feldman MW (2014) On the stability of the Bayenv method in assessing human SNP-environment associations. Hum Genomics 8: 1 10.1186/1479-7364-8-1 24405978 PMC3896655

[74] Kalnay E, Kanamitsu M, Kistler R, Collins W, Deaven D, et al (1996) The NCEP/NCAR 40-Year Reanalysis Project. Bulletin of the American Meteorological Society 77: 437–471. 10.1175/1520-0477(1996)077%3C0437:TNYRP%3E2.0.CO;2

