# Supplementary material for: Local Adaptation of Sun-Exposure-Dependent Gene Expression Regulation in Human Skin
Source: PLoS Genet. 2016 Oct 19;12(10):e1006382. doi: 10.1371/journal.pgen.1006382 (PMC5070784; doi:10.1371/journal.pgen.1006382)
Supplement: S1 Text — (PDF) [file pgen.1006382.s001.pdf]

S1 Text

Comparison of sun-exposure differential expression between ancestries

The skin samples were obtained from individuals with diverse ancestry, allowing us to examine whether ancestry affects the difference in gene expression due to sun-exposure. After testing for differential expression due to sun-exposure in each group independently, we observed significantly correlated effects (Spearman rho = 0.50, $p < 10^{-15}$, S11B Fig). We then tested each gene for evidence of ancestry-specific sun-exposure differential expression (see Methods). Only four transcripts (ENSG00000225972.1, ENSG00000237973.1, ENSG00000134927.3, ENSG00000267519.2) were found to be significant at a FDR of 0.01 using the likelihood ratio test. Two of these transcripts are pseudogenes, one is an uncharacterized long non-coding RNA, and the other is a transcript from the gene, *TCN1*, which encodes for the protein haptocorrin. Increased expression of *TCN1* has been observed in response to epidermal skin wounds (Roupe et al. 2010, Nuutila et al. 2012). In the GTEx skin data, *TCN1* is expressed higher in European ancestry individuals after sun exposure - with no evidence of difference in the African ancestry individuals - suggesting a larger stress response in those with European ancestry (S13 Fig). Overall, however, there is a paucity of ancestry-specific sun-exposure differential expression, likely because of the small number of individuals with African ancestry.
