# Supplementary material for: Local Adaptation of Sun-Exposure-Dependent Gene Expression Regulation in Human Skin
Source: PLoS Genet. 2016 Oct 19;12(10):e1006382. doi: 10.1371/journal.pgen.1006382 (PMC5070784; doi:10.1371/journal.pgen.1006382)
Supplement: S2 Text — (PDF) [file pgen.1006382.s002.pdf]

S2 Text

<u>Simulation to demonstrate independence of effect-size test and differential ASE</u>

For each simulation, both sample types (SE and NSE, e.g.) were given a starting eQTL effect size sampled from norm(0,20), with norm(N,S) indicating a normal distribution with mean N and deviation S. To simulate a exposure-specific eQTL, the eQTL effect size for one sample type was multiplied by a factor randomly sampled from a uniform distribution between 1.1 and 2.0. To simulate the null, i.e. the absence of an exposure-specific eQTL, the eQTL effect size was not adjusted for either sample type. The allelic expression for each allele in each sample was simulated based on the eQTL effect size by max(10 , norm(100+E*D, E0)), where E is the eQTL effect size for the sample type, D is an indicator variable of whether the allele is upregulated, and E0 is the non-inflated eQTL effect size. The overall expression for a gene (used in the effect-size test) is the sum of the expression of the two alleles. The genotype data was simulated by sampling genotypes to obtain the same allele frequency for each exposure-type, where the allele frequency is randomly sampled between 0.05 to 0.95 (MAF > 0.05). 250 individuals were simulated for each exposure-type. Varying the parameters of the simulations produced similar results.
