# Supplementary figures and images for: Local Adaptation of Sun-Exposure-Dependent Gene Expression Regulation in Human Skin
Source: PLoS Genet. 2016 Oct 19;12(10):e1006382. doi: 10.1371/journal.pgen.1006382 (PMC5070784; doi:10.1371/journal.pgen.1006382)

S1 Fig

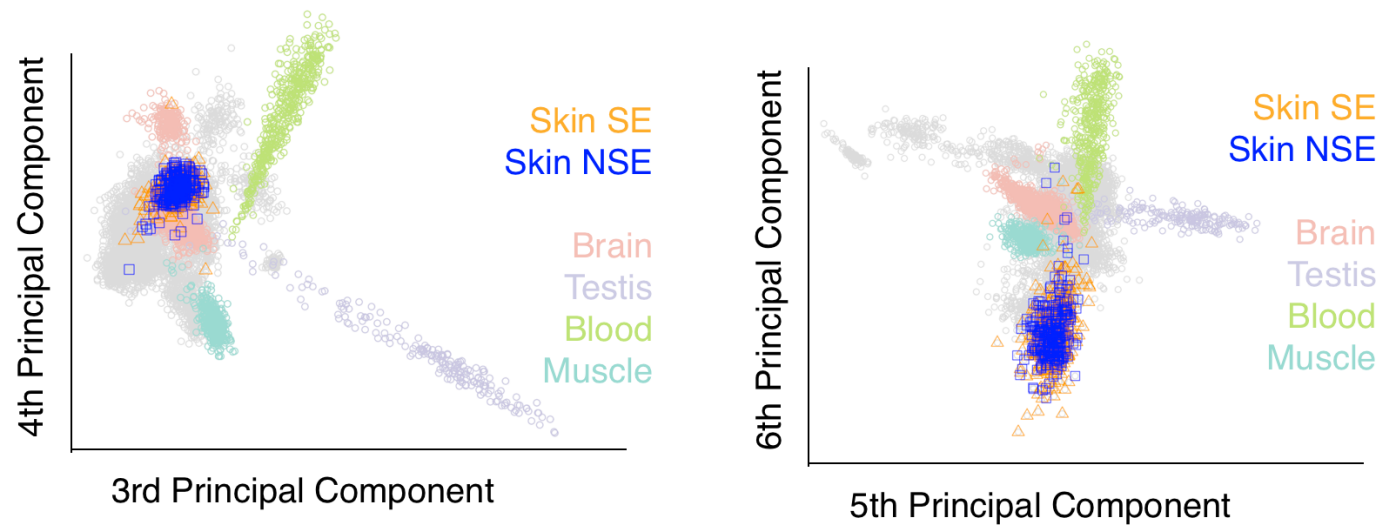

Supplement: S1 Fig — (PDF) [file pgen.1006382.s003.pdf]

S2 Fig

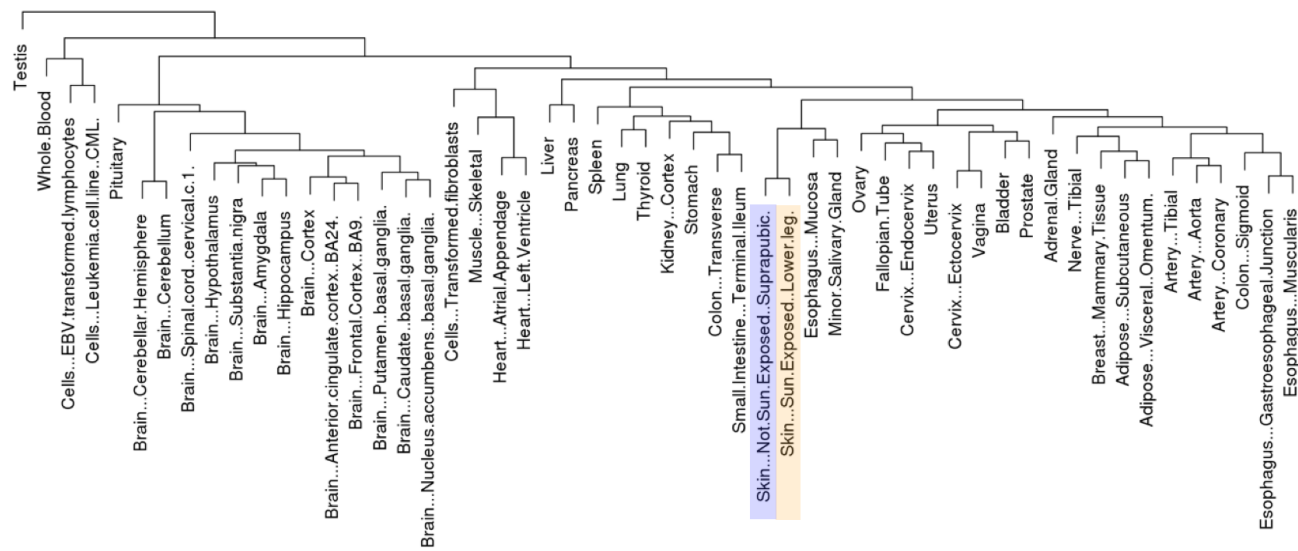

Supplement: S2 Fig — (PDF) [file pgen.1006382.s004.pdf]

S3 Fig

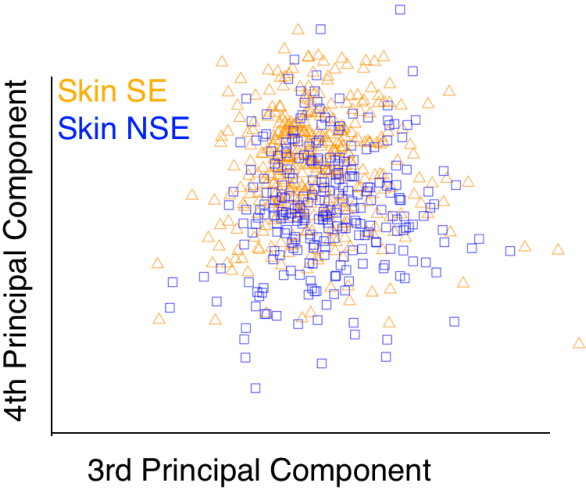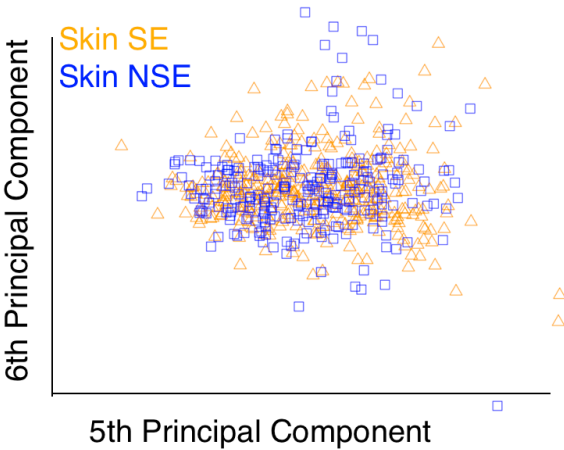

Supplement: S3 Fig — (PDF) [file pgen.1006382.s005.pdf]

S4 Fig

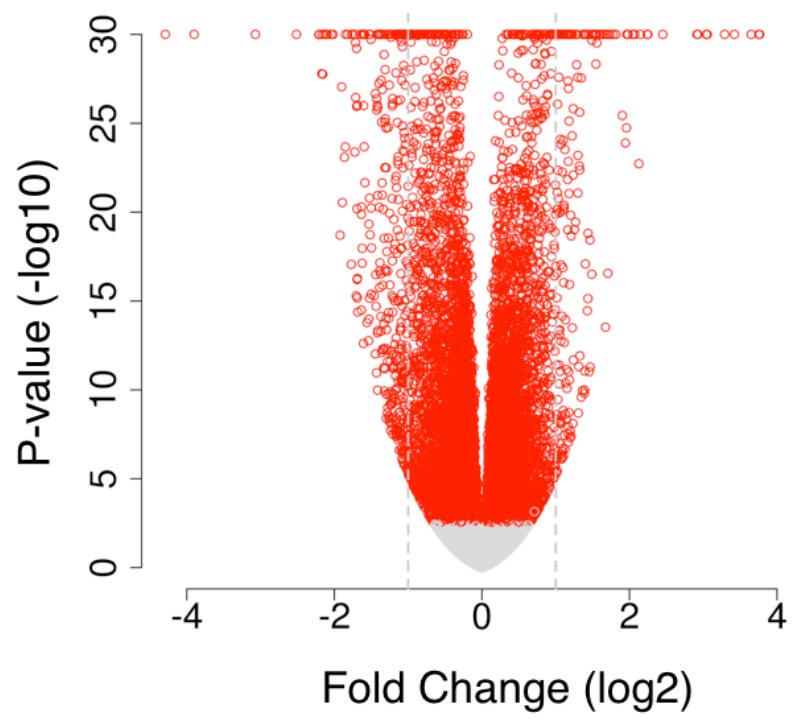

Supplement: S4 Fig — (PDF) [file pgen.1006382.s006.pdf]

S5 Fig

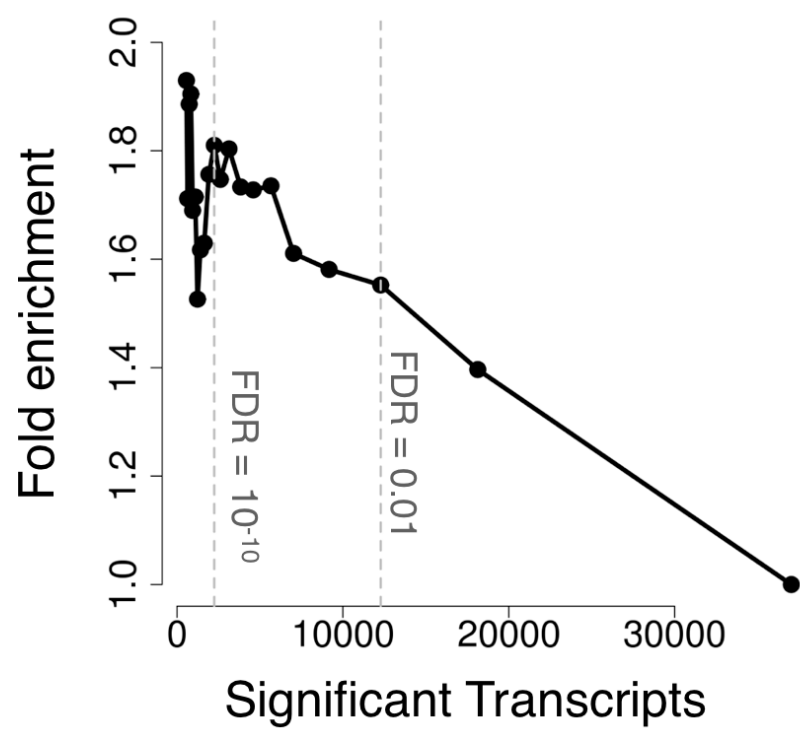

Supplement: S5 Fig — (PDF) [file pgen.1006382.s007.pdf]

S6 Fig

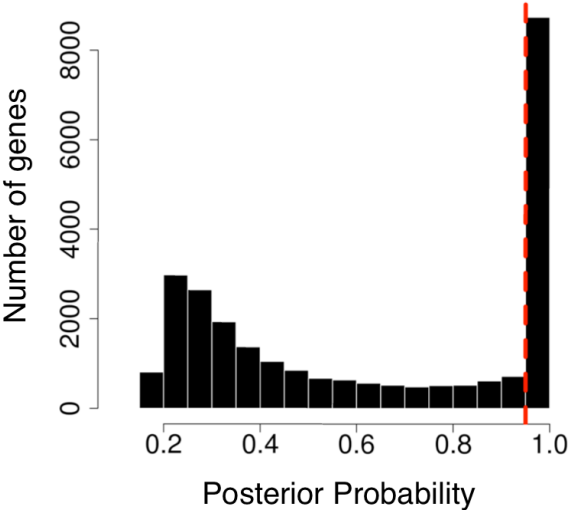

Supplement: S6 Fig — 8739 genes have > 0.95 posterior probability marked by the red dotted line. (PDF) [file pgen.1006382.s008.pdf]

# S7 Fig

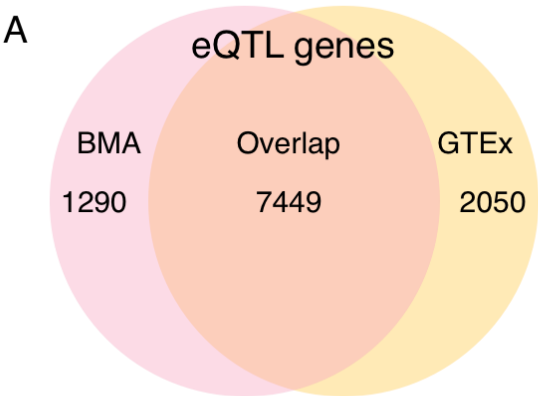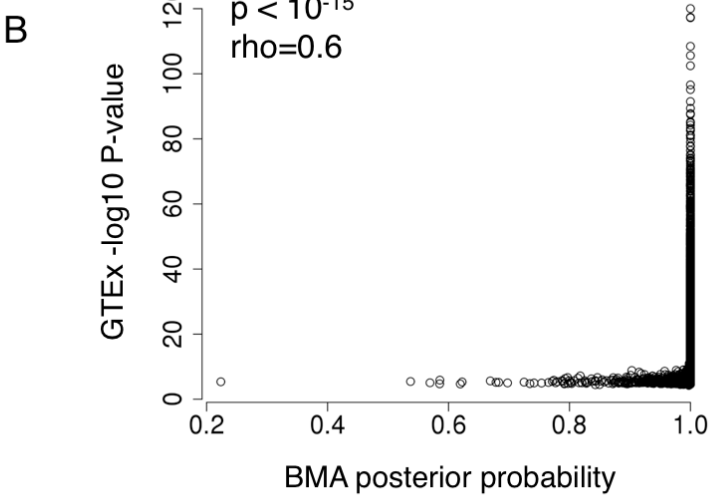

Supplement: S7 Fig — (A) Skin eQTLs from our BMA analysis overlap with skin eQTLs from GTEx. (B) GTEx -log10 P-values of the eQTL correlate significantly with the posterior probability from in BMA. Only eQTLs found in the GTEx analysis were used in this correlation. (PDF) [file pgen.1006382.s009.pdf]

# S8 Fig

A

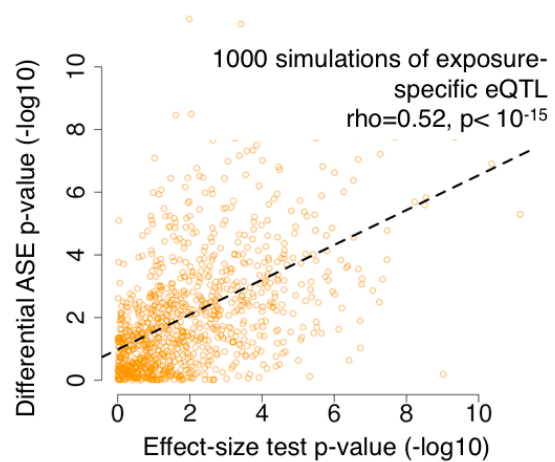

B

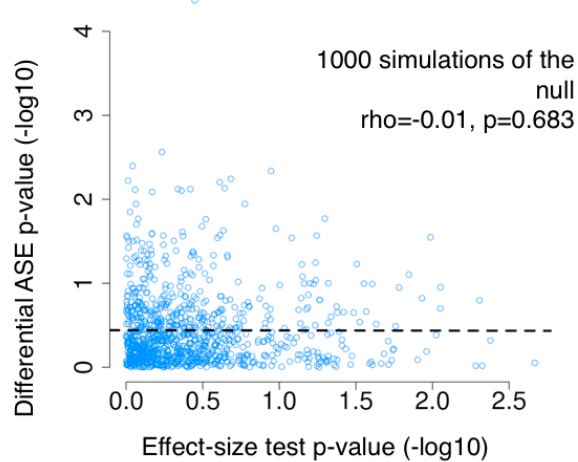

Supplement: S8 Fig — (A) 1000 simulations of an exposure-specific eQTL indicate that the differential ASE test and effect-size test have significantly correlated results. This indicates that both methods can be used to detect the signal of differential cis-regulation. (B) 1000 simulations of the null (absence of an exposure-specific eQTL) indicate that the effect-size test and the differential ASE analysis do not show a correlated result, even though both tests use data derived from the same simulation. This indicates that the differential ASE analysis and the effect-size test are independent tests. See S2 Text for details on simulations. (PDF) [file pgen.1006382.s010.pdf]

# S9 Fig

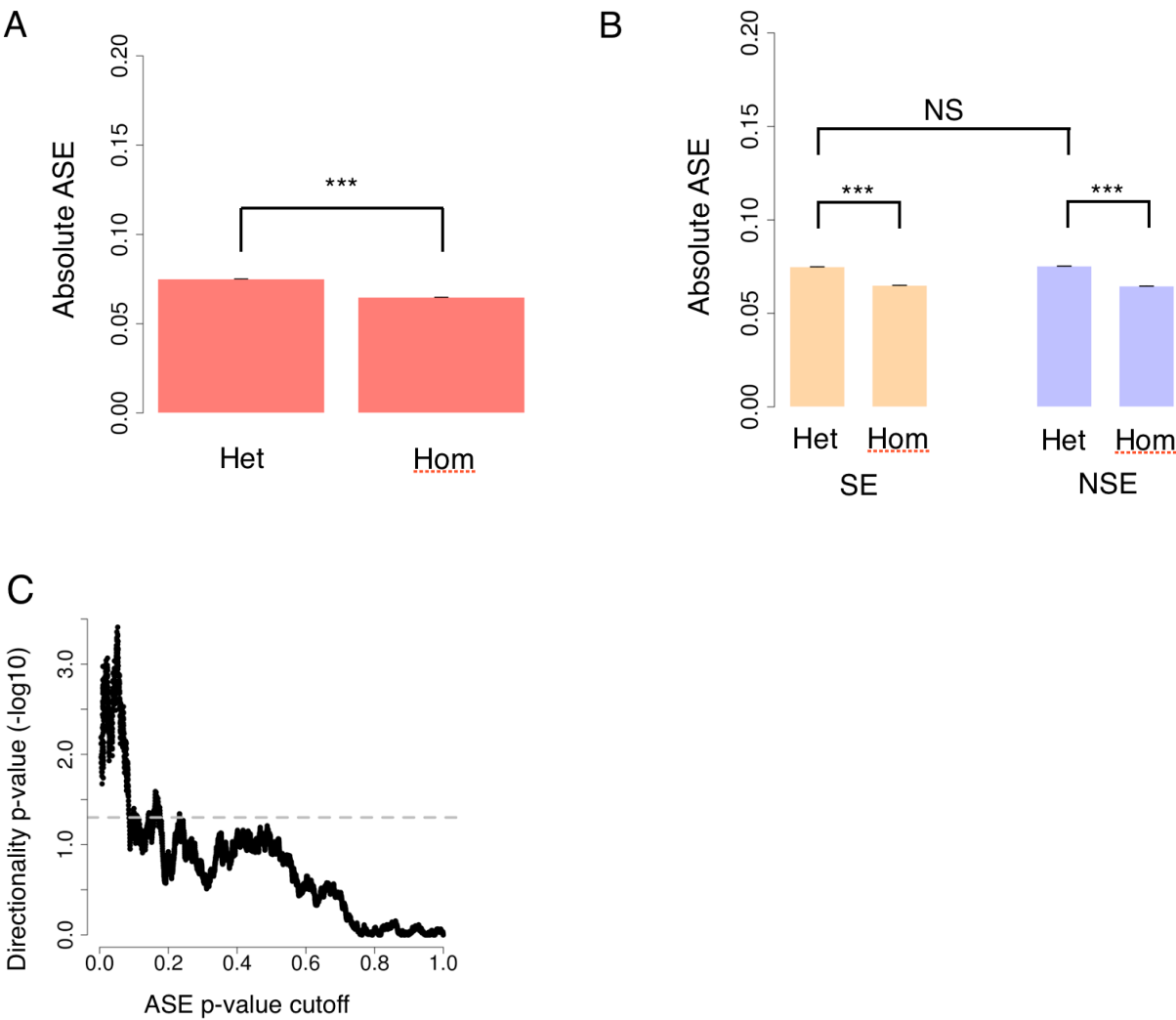

Supplement: S9 Fig — (A) Absolute ASE of all cis-eQTLs, separated by measurements when the individual was homozygous (Hom) at the eQTL or heterozygous at the eQTL (Het). Significance was assessed by Wilcoxon rank-sum test. *** marks p < 10−15. Note that the magnitude of the effect size is similar to other ASE analyses [69] (B) Absolute ASE of all cis-eQTLs separated by genotype at the eQTL and exposure-type. Significance was assessed by Wilcoxon rank-sum test. NS marks the p >0.05. *** marks p < 10−15. (C) Significance of concordant directionality between the effect-size test and the differential ASE analysis as a function of the p-value cutoff in the differential ASE analysis. Directionality p-value is calculated by the two-sided binomial test, where the expected fraction of concordance is set at the concordance of all genes (0.36). (PDF) [file pgen.1006382.s011.pdf]

S10 Fig

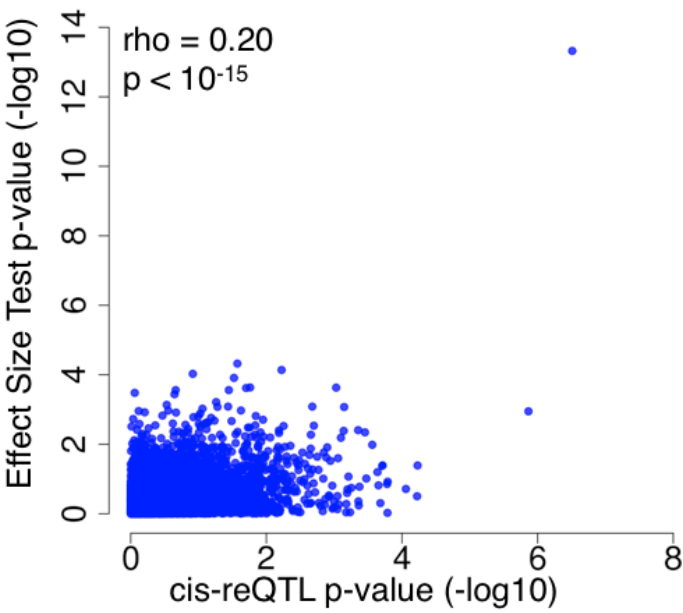

Supplement: S10 Fig — (A) Effect-size p-value vs cis-reQTL p-value for all associations that were tested in both analyses (1 per gene). P-value is calculated from the Spearman correlation. (PDF) [file pgen.1006382.s012.pdf]

S11 Fig

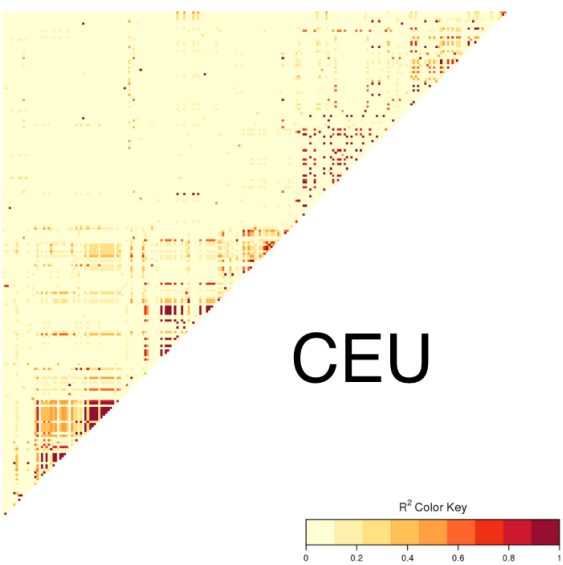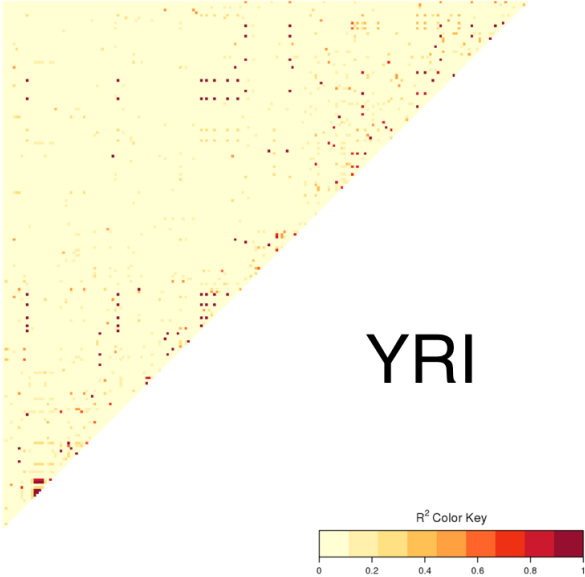

Supplement: S11 Fig — The cis-reQTL found in this study (rs12653176) is located at the bottom and the known selected non-synonymous SNP (rs16891982) is located at the top. The distance between the two SNPs is 278kb. (PDF) [file pgen.1006382.s013.pdf]

S12 Fig

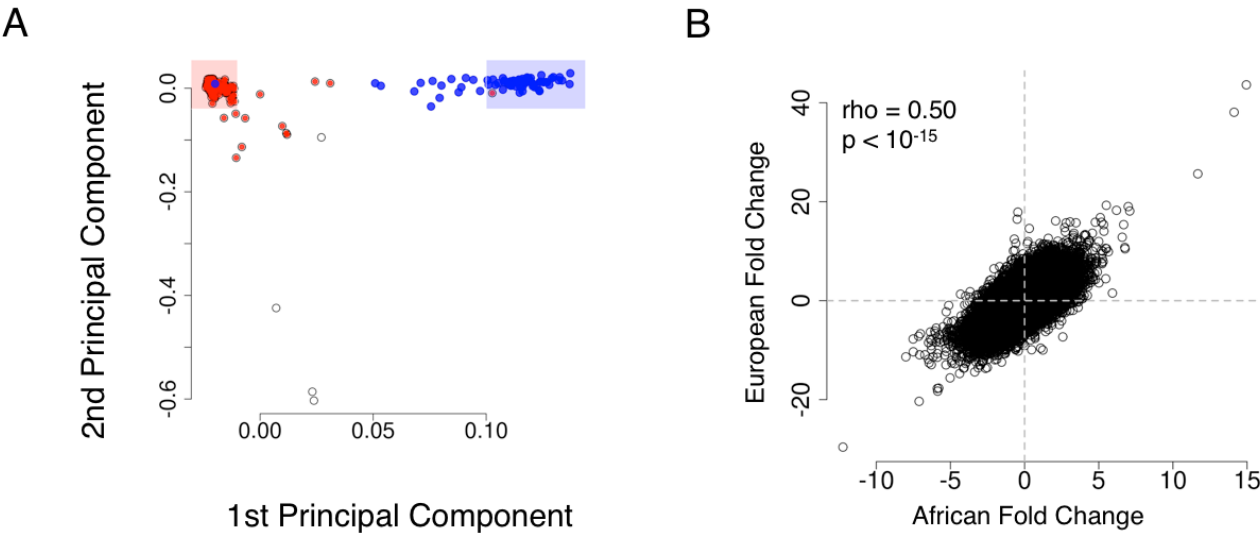

Supplement: S12 Fig — (A) Principal components of genotype (calculated by GTEx) separate individuals into ancestry groups. Red is used to mark the reported race “White”. Blue marks the reported race of “Black or African American”. Empty circles represent other reported race. Red shaded region demarcate the designated principal component boundaries in this study for the European ancestry population resulting in 377 genotyped individuals. Blue shaded region demarcates the African ancestry population, resulting in 68 genotyped individuals. (B) The fold change, log (SE / NSE), of all genes in the European ancestry individuals and the African ancestry individuals. P-value was calculated based on the asymptotic approximation of the Spearman correlation (rho). (PDF) [file pgen.1006382.s014.pdf]

S13 Fig

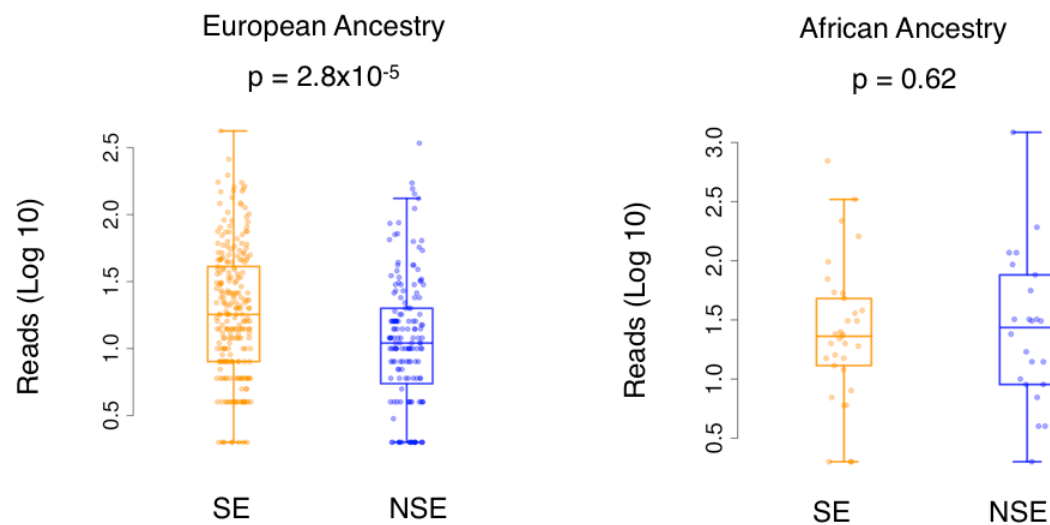

Supplement: S13 Fig — Even with the substantially lower power in the African ancestry individuals, we see a significant difference in differential expression in this gene at a Benjamini-Hochberg FDR < 0.01 (p = 5.6x10^-7^, likelihood ratio test). (PDF) [file pgen.1006382.s015.pdf]
